# Duplicate prescriptions—proposal of a clinically oriented categorisation

**DOI:** 10.1007/s00228-021-03143-8

**Published:** 2021-04-23

**Authors:** Johannes Heck, Benjamin Krichevsky, Dirk O. Stichtenoth, Christoph Höner zu Siederdissen, Olaf Krause

**Affiliations:** 1 Institute for Clinical Pharmacology, Hannover Medical School, Hannover, Germany; 2 Medical Service of the German Armed Forces, Kiel, Germany; 3 Drug Commissioner of Hannover Medical School, Hannover, Germany; 4 Emergency Department, Hannover Medical School, Hannover, Germany; 5 Institute for General Medicine, Hannover Medical School, Hannover, Germany

Sir,

In clinical routine, we frequently encounter duplicate prescriptions. Duplicate prescriptions pose a risk factor for the development of adverse drug reactions and may increase healthcare expenditures. Literature on the prevalence of duplicate prescriptions is abundantly available [1–6]. However, to the best of our knowledge, a widely accepted definition of “duplicate prescription” has not been established to date. Van Leeuwen and colleagues, for instance, merely “define a duplicate prescription as the concurrent use of two drugs of the same class to treat the same condition” [2]. This definition neglects two important aspects. First, two drugs of different pharmacological classes, such as the combination of a proton pump inhibitor and a histamine H_2_ receptor antagonist, may also be considered a duplicate prescription. Second, two drugs of the same pharmacological class used in different therapeutic indications, such as the combination of diphenhydramine for nausea and vomiting and doxylamine for sleep disturbances, also constitute a duplicate prescription.

From a clinical viewpoint, we suggest to differentiate between appropriate and potentially inappropriate duplicate prescriptions (Table 1). In analogy to potentially inappropriate medications (PIMs), i.e. drugs that are considered unsuitable for elderly people [7], we propose the term potentially inappropriate duplicate prescriptions (PIDPs). Deciding whether a duplicate prescription is appropriate or potentially inappropriate represents a challenging task that requires both in-depth clinical and pharmacological knowledge. The evaluation should be performed by a physician or preferably multiple physicians from different specialties for an increased reliability [8, 9], and should always consider a patient’s individual circumstances such as medication history, comorbidities and patient preferences.

**Table 1:** Proposal of a differentiation of duplicate prescriptions in appropriate duplicate prescriptions (ADPs) and potentially inappropriate duplicate prescriptions (PIDPs). PIDPs are further subdivided into three grades, with higher grades indicating an increasing degree of inappropriateness. *ACE*, angiotensin-converting enzyme; *ARB*, angiotensin-receptor blocker; *HMG-CoA*, hydroxymethylglutaryl coenzyme A; *PPI*, proton pump inhibitor; *SGLT2*, sodium–glucose cotransporter type 2

Duplicate prescriptions				
	Appropriate duplicate prescriptions (ADPs)	Potentially inappropriate duplicate prescriptions (PIDPs)		
Grade	—	1	2	3
Description	Two drugs with therapeutically desired synergistic effects (that is, established combination treatments)	Two drugs with overlapping or comparable pharmacodynamics	Two drugs of the same therapeutic class (that is, targeting the same molecular structure)	Two times the same drug (exceeding the recommended maximum daily dose)
Examples	HMG-CoA reductase inhibitor + ezetimibe	ACE inhibitor + ARB	Two different opioid analgesics (e.g. buprenorphine + hydromorphone; oxycodone + tramadol)	Hydrochlorothiazide both as single agent and as partner in an antihypertensive combination product
	Metformin + SGLT2 inhibitor	ACE inhibitor + aliskiren	Hydrochlorothiazide + chlorthalidone	Valsartan both as single agent and in sacubitril–valsartan
	Opioid analgesic + non-opioid analgesic	PPI + H_2_ receptor antagonist	Ibuprofen + diclofenac	Paracetamol both as single agent and in an acetylsalicylic acid–paracetamol–caffeine combination product
	ACE inhibitor + thiazide diuretic	Paracetamol + ibuprofen	Lorazepam + diazepam	Ibuprofen both as single agent and in an ibuprofen–caffeine combination product
	Acetylsalicylic acid + clopidogrel	Ibuprofen + metamizole	Amlodipine + lercanidipine	Diclofenac both as single agent and in a diclofenac–misoprostol combination product
	Loop diuretic + thiazide diuretic	Doxylamine + zopiclone	Diphenhydramine + doxylamine	Codeine both as single agent and in a paracetamol–codeine combination product

In internal medicine, myriad examples of appropriate duplicate prescriptions (ADPs), i.e. rational and established combination treatments, exist (corresponding indications in parentheses): hydroxymethylglutaryl coenzyme A reductase inhibitor (“statin”) + ezetimibe (hypercholesterolemia); opioid analgesic + non-opioid analgesic (postoperative pain); acetylsalicylic acid + P2Y_12_ receptor antagonist, e.g. clopidogrel (following coronary stent implantation); loop diuretic + thiazide diuretic (forced diuresis); combination of two antidiabetics of different pharmacological classes, e.g. metformin + sodium–glucose cotransporter type 2 inhibitor. This list is, of course, not exhaustive, and many more examples of ADPs can be thought of.

With regard to PIDPs, we propose a categorisation into three different grades, with higher grades indicating an increasing degree of inappropriateness (Table 1). We believe that our proposed categorisation allows a more subtle differentiation of PIDPs in comparison to previous publications on this topic.

Of note, our categorisation is a simplified model and therefore has certain limitations. Multiplicate (that is, ≥ triplicate) prescriptions are not part of our scheme. We may, for example, think of an elderly gentleman suffering from arterial hypertension who is being treated with a quadruplicate antihypertensive regimen consisting of amlodipine, bisoprolol, moxonidin and hydrochlorothiazide. Evaluation of such a complex therapy requires further knowledge about the patient’s comorbidities, comedication and previous medical history including adverse drug reactions and may be considered appropriate or inappropriate, depending on the clinical context. Such an evaluation is clearly beyond the scope of our categorisation.

In conclusion, we propose a differentiation of duplicate prescriptions into appropriate and potentially inappropriate duplicate prescriptions. Furthermore, we suggest subdividing PIDPs into three grades. We strongly encourage fellow physicians to question the appropriateness of duplicate prescriptions in clinical routine and to terminate PIDPs wherever possible, especially grade 3 PIDPs. As with all models, there may be exceptions from the rule. Specialist medications, such as antineoplastic or immunomodulatory agents, should not be discontinued without prior consultation of the prescribing physician.

We hope that the readership may find our proposal convincing and that the term PIDP and its subdivision into three grades will be adopted in future research projects about the clinically relevant topic of duplicate prescriptions.
